# Supplementary material for: Multi‐dataset identification of innovative feature genes and molecular mechanisms in keratoconus
Source: J Cell Mol Med. 2024 Sep 19;28(18):e70079. doi: 10.1111/jcmm.70079 (PMC11412914; doi:10.1111/jcmm.70079)
Supplement: Supplementary file 2 — Material S2. [file JCMM-28-e70079-s002.zip › 7.GSEA analysis/4.MYRF/KEGG_GLYCOSAMINOGLYCAN_BIOSYNTHESIS_KERATAN_SULFATE.html]

Details for gene set KEGG\_GLYCOSAMINOGLYCAN\_BIOSYNTHESIS\_KERATAN\_SULFATE[GSEA]

|  || Dataset | MYRF.group.cls#h\_versus\_l |
| Phenotype | group.cls#h\_versus\_l |
| Upregulated in class | l |
| GeneSet | KEGG\_GLYCOSAMINOGLYCAN\_BIOSYNTHESIS\_KERATAN\_SULFATE |
| Enrichment Score (ES) | -0.49291325 |
| Normalized Enrichment Score (NES) | -1.3741205 |
| Nominal p-value | 0.14020619 |
| FDR q-value | 0.24799658 |
| FWER p-Value | 0.904 |
Table: GSEA Results Summary

  

Fig 1: Enrichment plot: KEGG\_GLYCOSAMINOGLYCAN\_BIOSYNTHESIS\_KERATAN\_SULFATE      
 Profile of the Running ES Score & Positions of GeneSet Members on the Rank Ordered List

  

| SYMBOL | TITLE | RANK IN GENE LIST | RANK METRIC SCORE | RUNNING ES | CORE ENRICHMENT || 1 | CHST2 | na | 1424 | 0.408 | 0.0976 | No |
| 2 | CHST6 | na | 9945 | 0.097 | -0.1149 | No |
| 3 | B4GALT2 | na | 11157 | 0.076 | -0.1241 | No |
| 4 | B4GAT1 | na | 11493 | 0.070 | -0.1098 | No |
| 5 | B3GNT7 | na | 13722 | 0.041 | -0.1600 | No |
| 6 | ST3GAL1 | na | 19414 | -0.015 | -0.3188 | No |
| 7 | B4GALT4 | na | 23007 | -0.060 | -0.4020 | No |
| 8 | B4GALT1 | na | 24027 | -0.076 | -0.4054 | No |
| 9 | CHST4 | na | 27067 | -0.137 | -0.4464 | Yes |
| 10 | B4GALT3 | na | 27814 | -0.156 | -0.4150 | Yes |
| 11 | B3GNT2 | na | 28962 | -0.190 | -0.3835 | Yes |
| 12 | ST3GAL2 | na | 31066 | -0.267 | -0.3532 | Yes |
| 13 | FUT8 | na | 32277 | -0.335 | -0.2743 | Yes |
| 14 | CHST1 | na | 33482 | -0.441 | -0.1591 | Yes |
| 15 | ST3GAL3 | na | 34209 | -0.574 | 0.0150 | Yes |
Table: GSEA details [plain text format]

  

Fig 2: KEGG\_GLYCOSAMINOGLYCAN\_BIOSYNTHESIS\_KERATAN\_SULFATE      
 Blue-Pink O' Gram in the Space of the Analyzed GeneSet

  

Fig 3: KEGG\_GLYCOSAMINOGLYCAN\_BIOSYNTHESIS\_KERATAN\_SULFATE: Random ES distribution      
 Gene set null distribution of ES for **KEGG\_GLYCOSAMINOGLYCAN\_BIOSYNTHESIS\_KERATAN\_SULFATE**
